# Supplementary material for: MetaRibo-Seq measures translation in microbiomes
Source: Nat Commun. 2020 Jun 29;11:3268. doi: 10.1038/s41467-020-17081-z (PMC7324362; doi:10.1038/s41467-020-17081-z)
Supplement: Supplementary file 10 — Supplementary Data 7 [file 41467_2020_17081_MOESM10_ESM.zip › File2/Confidence_High_Taxonomy/405286_out.krona.html]

Javascript must be enabled to view this page.

members
magnitude
magnitudeUnassigned
count
unassigned
taxon
rank

405286\_out

3

2
superkingdom
3

976
phylum
3

class
200643
3

3
171549
order

171552
family
3

3
838
genus

3

SRS019808\_contig\_number\_18780SRS023715\_contig\_number\_23842SRS043667\_contig\_number\_23
165179
species
